# Stromal Expression of CD10 in Invasive Breast Carcinoma and Its Correlation with ER, PR, HER2-neu, and Ki67

**DOI:** 10.4061/2011/437957

**Published:** 2011-06-16

**Authors:** Vandana Puri, Manjula Jain, Shaji Thomas

**Affiliations:** ^1^Department of Pathology, Lady Hardinge Medical College & Smt. Sucheta Kriplani Hospital, New Delhi 110001, India; ^2^Department of Surgery, Lady Hardinge Medical College & Smt. Sucheta Kriplani Hospital, New Delhi 110001, India

## Abstract

CD10 is a cell surface zinc-dependent endopeptidase, which degrades many bioactive peptides. CD10 expression in tumour stroma is associated with biological aggressiveness of many epithelial malignancies. *To date, only one study has correlated with expression of CD10 with well-known prognostic markers of breast, that is, ER, PR, HER2-neu, and tumour grade; however, its correlation with ki67 is still not studied*. The aim of this study is to evaluate stromal CD10 expression in breast carcinoma and to examine its correlation with ER, PR, HER2-neu, and Ki67. *Methods and Results*. CD10 expression in fifty patients was assessed by immunohistochemistry and scored as negative, weak and strong. CD10 was found to be positive in stroma of 40/50 (80%) cases. Stromal CD10 showed positive correlation with tumour grade, HER2-neu (*P* = .000), and ki67 (*P* = .027), negative correlation with ER and PR. *Conclusions*. Hence CD10 expression correlated strongly with well-established negative prognostic markers, that is, HER2-neu and ki67 positivity, ER/PR negativity, and higher tumour grade, thus indicating that CD10 can be used as independent marker indicating poor prognosis and can be used as target for the development of novel therapies.

## 1. Introduction

Breast cancer is one of the commonest cancers among women globally_._ Each individual tumour varies with respect to invasive and metastatic potential as well as growth rate. The prognosis of a given lesion is related to member and types of oncogene activated. Various markers have been used to study the oncogene expression or amplification. The clinical course of the neoplasm can be altered by directing therapy against overexpressed oncogenes. Well-established prognostic factors including stage of tumour, histological grade, lymph node status, ER/PR status, and HER2-neu are routinely studied in every case of breast cancer. Stromal markers are now emerging as novel markers in assessing the prognosis of invasive breast cancer and have not been studied extensively till date. Stroma plays a key role in modulating tumour invasion and metastasis. A better understanding of stromal contribution to cancer progression will identify specific signals that promote growth, dedifferentiation, invasion, and ectopic survival of tumour cells and eventually result in the identification of new therapeutic targets for future treatment [1]. This justifies the study of new stromal marker CD10 in the prognosis of invasive breast carcinoma.

CD10 is a zinc-dependent metalloproteinase that has been called common acute lymphoblastic antigen (CALLA). It is frequently expressed in bone marrow lymphoid stem cells, pro-B lymphoblasts, mature neutrophils, various lymphoma subtypes, renal cell carcinoma and endometrial stromal sarcoma. Several reports indicated that stromal CD10 expression is associated with biological aggressiveness in various epithelial malignancies [2–6]. 

To date, only one* study has correlated the expression of CD10 with well-known prognostic markers of breast carcinoma; however, its correlation with* Ki67 is still not studied. The aims of this study were, (1) to estimate the frequency of expression of stromal CD10 in invasive breast carcinomas and (2) to assess prognostic significance of stromal CD10 expression and its correlation with known prognostic markers of breast carcinoma.

## 2. Materials and Methods

The present study was conducted in the department of pathology and surgery.

Cases of breast lump were referred to pathology department from surgery clinics for FNAC during the study period, that is, from November 2007 to March 2009. Trucut biopsy was performed on cytologically proven cases of breast carcinoma, and the following parameters were noted:

1. age,
2. family history,
3. clinical presentations-lump/pain/nipple discharge,
4. clinical TNM staging.

Study group comprised fifty cases of breast carcinoma diagnosed on trucut biopsy. Trucut biopsy was performed on patients using 14-gauge needle of length 3 inches, under full aseptic precautions, using local anaesthesia. Tissue from trucut biopsy was processed, paraffin blocks were prepared, and subsequently six slides were cut from each.

**Figure 1:**
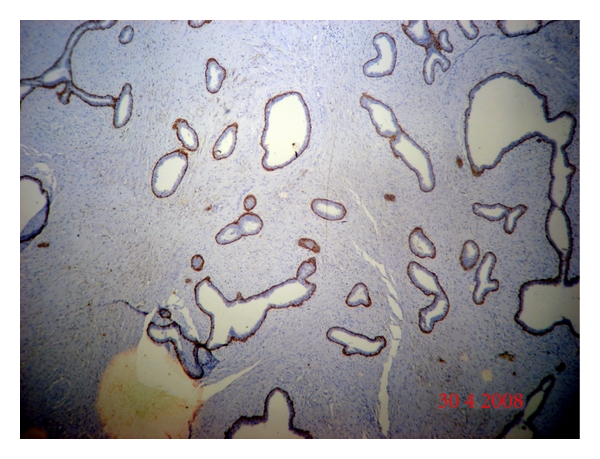
Fibroadenoma in this figure shows highlighted myoepithelial cells by CD10. There was no expression of CD10 in normal ductal cells, fibroblasts.

Slide1: Stained with haematoxylin and eosin.
Slide 2: Immunohistochemistry for estrogen receptor (Diagnostic biosystems ER, 1D5 clone), dilution 1 : 50.
Slide 3: Immunohistochemistry for progesterone receptor (Diagnostic biosystems PR (hPRa2 + hPRa3 clone), dilution 1 : 50.
Slide 4: Immunohistochemistry for HER2-neu (Diagnostic biosystems HER2-neu, CD11 clone). Dilution 1 : 50.
Slide 5: Immunohistochemistry for CD10 (Diagnostic biosystems CD10 (56C6 clone), dilution 1 : 30. Sections of fibroadenoma were put up as control (Figure 1).
Slide 6: Immunohistochemistry for ki-67 Ki67 (MIB-1 clone) DAKO, mouse monoclonal antibody, dilution 1 : 500.

LSABC kit was used as the detection system. ER and PR stain scoring was done by quick score. HER2-neu scoring was done as in (Table 1) CD10 scoring was done as in (Table 2).

Statistical analysis was done, and *P* values less than  .05 were considered significant.

## 3. Results

**Figure 2:**
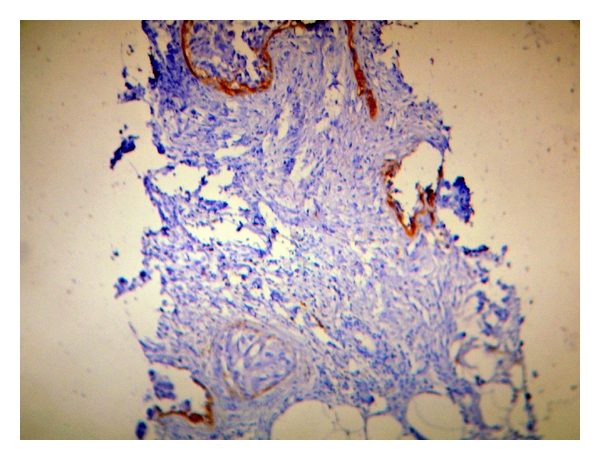
Nonneoplastic myoepithelial cells, whenever present, were used as built-in positive control.

Mean age of patients was 48.5 years (range 30–80 years). Most of our cases belonged to T4 category (21/50; 42%) followed by T2 category (15/50; 30%) and T1 category (14/50; 28%). Infiltrating ductal carcinoma, not otherwise specified (NOS), the comprised majority of our study population (47/50; 94%) followed by two cases (4%) of mucinous carcinoma and one case (2%) of infiltrating ductal carcinoma with extensive in situ component. Bloom and Richardson grading was performed on all cases except one, which was IDC with extensive in situ component. Most patients 26/49 (53.06%) in our study belonged to grade 3, while 17/49 (34.69%) belonged to grade 2 and 6/49 (12.24%) belonged to grade 1. CD10 immunostaining was done on all 50 cases. No stromal expression was detected in the normal breast although the Nonneoplastic myoepithelial cells, whenever present, served as a built-in positive control for CD10 (Figure 2). There was no expression of CD10 in normal ductal cells, fibroblasts, and adipose cells. The staining was scored as negative, weak, and strong as described in Table 2.

**Figure 3:**
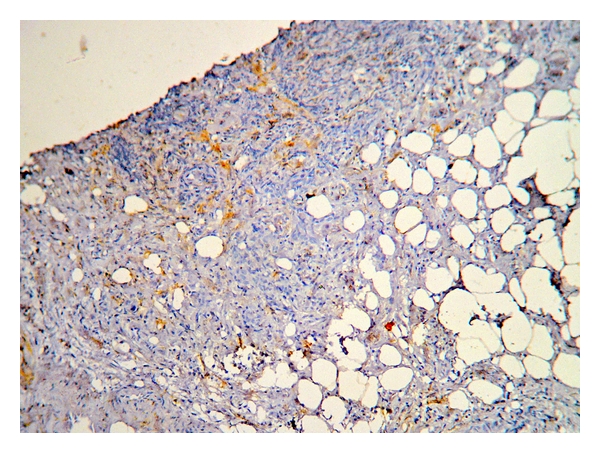
Weak CD10 stromal staining.

**Figure 4:**
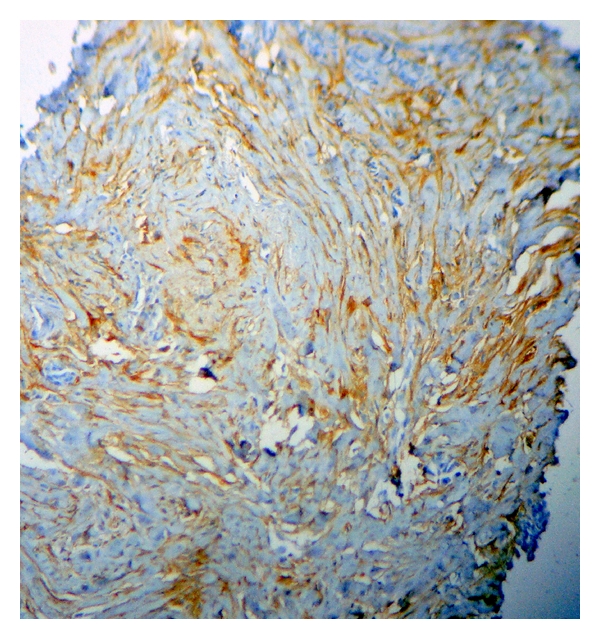
Strong CD10 stromal staining.

CD10 was found to be positive in 80% of cases (40/50), out of which 16 (40%) showed weak immunoreactivity (Figure 3) whereas strong positivity (Figure 4) was observed in 24 cases (60%) which included one case of IDC with extensive in situ component. Extensive in situ component was clearly highlighted by strong positivity of CD10 in myoepithelial cells. The same case also showed foci of stromal CD10 positivity indicating invasion.

### 3.1. Expression of Stromal CD10 in Breast Cancer and Correlations with Other Clinicopathological Data

A positive correlation was observed between CD10 and tumour grade, however it was not statistically significant (*P* = .139). Percentage positivity of strong CD10 increased from 50% to 64.71% in T2 to T4 category, but it could not reach statistical significance (*P* = .558). With increasing CD10 positivity from weak to strong, ER negativity increased from 43.75% to 87.5%. (*P* = .188) (Table 3).

Similarly CD10 was found to have a negative correlation with PR however it was not statistically significant. A strongly positive statistical significance was found between CD10 positivity and HER2-neu (*P* = .000) (Table 4).

 A positive statistical significance was found between CD10 positivity and Ki67 (*P* = .027) (Table 5).

## 4. Discussion

CD10 is a cell surface zinc-dependent metalloproteinase. Matrix metalloproteinases are a family of metallopeptidases that cleave the protein components of extracellular matrix and thereby play a central role in tissue remodelling. A better understanding of stromal contribution to cancer progression will identify specific signals that promote growth, dedifferentiation, invasion, and ectopic survival of tumour cells and eventually result in identification of new therapeutic targets for future treatment [1]. According to the study by Makretsov et al. [4], stromal CD10 is positive in 79% of patients, with weak immunoreactivity in 40% cases whereas strong positivity was observed in 39% cases. In our study also, CD10 was positive in 80% of patients similar to the above study with strong stromal positivity in 60% of cases and weak immunoreactivity in 40% of cases. More cases of strong CD10 were present in our study as most of our patients had grade 3 tumours whereas in a study by Makretsov et al. the majority of the patients had grade 2 tumours. In a study by Iwaya et al. [3], no statistically significant correlation was observed between CD10 and tumour grade (*P* = .1613). According to study by Makretsov et al., percentage positivity of strong CD10 increased from 29% to 59% in grade 1 to grade 3. Our results also showed very similar trends. One case in our study was of IDC with extensive in situ component. Extensive in situ component was clearly highlighted by strong positivity of CD10 in myoepithelial cells. The same case also showed foci of strong stromal CD10 positivity indicating invasion. Martinez et al. proved in their study that there is a relationship between the presence of extensive intraductal component and the risk of local recurrence for patient with invasive breast cancer treated with conservative surgery and radiation surgery [7]. Extensive intraductal component constitutes a very important predictive factor for local breast recurrence. Hence CD10 can help as a useful marker in identifying invasion in problematic cases with in situ component. Makretsov et al. [4] showed statistically significant correlation between strong CD10 staining and ER negativity (*P* − .002). Similarly in our study, CD10 was found to have good negative correlation with ER; however, it was not statistically significant probably due to less number of cases in our study. Makretsov et al. [4], found no statistical significance was found between stromal CD10 expression and PR status (*P* > .05).This is in accordance with our study, where CD10 was found to have good negative correlation with PR, however it was not statistically significant (*P* > .05). A strongly positive statistical significance was found between CD10 positivity and HER2-neu (*P* = .000) in our study. On the contrary Makretsov et al. [4], found no statistically significant correlation between stromal CD10 expression and HER2-neu status of tumour (*P* > .05). However since only one study has given the correlation with stromal CD10 expression and HER2-neu expression of tumour till date, further studies are needed to firmly establish the correlation. A positive statistical significance was found between CD10 positivity and Ki67 (*P* = .027) No study till date has correlated with stromal CD10 and ki67.

To conclude CD10 expression correlated strongly with well-established negative prognostic marker that is, HER2-neu and ki67 positivity, ER/PR negativity, and higher tumour grade. thus indicating, CD10 can be used as independent marker indicating poor prognosis and can be used as target for development of novel therapies.

Thus, we suggest that core needle biopsy should be an important part of preoperative workup and along with the traditional panel of markers, CD10 can be put up routinely as a prognostic marker in all breast cancer patients.

## Figures and Tables

**Table 1:** HER2-neu scoring.

Staining pattern	Score	HER2-neu overexpression
No staining or membrane staining <10% tumor cells	0	negative
Faint/perceptible membrane staining in >10% tumor cells	1	negative
Weak to moderate complete membrane staining in >10% tumor cells	2	weak
Strong complete membrane staining in >10% tumor cells	3	strong

**Table 2:** CD10 scoring.

Score	CD10 Staining
Negative	<10% stromal positive cells/core
Weak	10–30% stromal positive cells/core
Strong	>30% stromal positive cells/core

**Table 3:** Correlation of CD10 with ER.

CD10	ER Negative	ER Positive	Total
Negative	7 (70%)	3 (30%)	10
Weak	7 (43.75%)	9 (56.25%)	16
Strong	21 (87.5%)	3 (12.5%)	24

Total	35	15	50

**Table 4:** Correlation of CD10 with HER2-neu.

HER2-neu	CD10 negative	CD10 positive		Total
		Weak	Strong	
Negative	8	9	3	20
Score 2	1	3	4	8
Score 3	1	4	17	22

Total	10	15	25	50

*P* = .000.

**Table 5:** Correlation of CD10 with Ki67.

CD10	Ki67 negative	Ki67 positive	Total
Negative	2	8	10
Weak	1	14	15
Strong		25	25

Total	3	47	50

*P* = .027.
